# Prevalence of oral rehydration solution use and its determinants in the treatment of diarrhea among under-five children in sub-Saharan Africa

**DOI:** 10.1371/journal.pone.0303193

**Published:** 2024-05-03

**Authors:** Sulaimon T. Adedokun, Sanni Yaya

**Affiliations:** 1 Department of Demography and Social Statistics, Obafemi Awolowo University, Ile-Ife, Nigeria; 2 School of International Development and Global Studies, University of Ottawa, Ottawa, Canada; 3 The George Institute for Global Health, Imperial College London, London, United Kingdom; 4 Faculty of medicine, University of Parakou, Parakou, Benin; EPICENTRE, CAMEROON

## Abstract

**Background:**

Diarrhea is the second leading cause of under-five deaths claiming half a million children every year. Most of these deaths occurred in sub-Saharan Africa and South Asia. Oral rehydration solution (ORS) has been described as the most effective treatment of diarrhea. However, only 36% of children with diarrhea received ORS in sub-Saharan Africa. This study examined the factors associated with ORS use for children with diarrhea in the sub-region.

**Methods:**

Demographic and Health Surveys (DHS) data sets of 31 countries in sub-Saharan Africa were used in this study. The data involved 30,102 under-five children with diarrhea. The multivariable analysis involved binary logistic regression.

**Results:**

Prevalence of ORS use was 38% in sub-Saharan Africa with countries such as Namibia (71.8%), Zambia (66.4%) and Malawi (63.8%) having the highest rates. Use of ORS was most common among children whose mothers had secondary or higher education (45%), were exposed to media (41%) and attended antenatal care (41%). ORS use was significantly associated with secondary or higher education **(**OR = 1.63; 95%CI: [1.47–1.81]; p<0.001), exposure to media (OR = 1.17; 95%CI: [1.07–1.27]; p<0.001), antenatal care attendance (OR = 2.33; 95%CI: [1.08–1.27]; p<0.001), child’s age (OR = 1.46; 95%CI: [1.35–1.59]; p<0.001), child’s size at birth (OR = 1.08; 95%CI: [1.00–1.17]; p<0.05), household size (OR = 1.28; 95%CI:[1.06–1.54]; p<0.05) and source of drinking water (OR = 1.18; 95%CI: [1.09–1.29]; p<0.001).

**Conclusion:**

This study revealed a 38% prevalence of ORS use during diarrhea episodes in sub-Saharan Africa. This is low as it is less than the 44% recorded for developing countries as a whole. While this study emphasises the need for a further study on effects of severity of diarrhea on ORS use and factors determining differences in ORS use among countries, it also calls for interventions that will increase use of ORS is sub-Saharan Africa. Such interventions should include increase in literacy rate among girls and women, increase in the proportion of women with access to media, involvement of health workers in programmes that would promote antenatal care utilization among women at community level and provision of social amenities like pipe-borne water.

## Background

Diarrhea disease is described as the second leading cause of death among under-five children, claiming the lives of half a million children every year [1]. Most of these deaths occurred in sub-Saharan Africa and South Asia [2]. Although it is highly recommended that diarrhea risk should be reduced through interventions such as safe drinking-water, use of improved sanitation and hand-washing with soap, emphasis has been placed on the use of oral rehydration solution (ORS) in the treatment of diarrhea [1]. Oral rehydration solution is a mixture of clean water, salt and sugar which is absorbed in the small intestine and replaces the water and electrolytes lost in the stool [1]. Its efficacy in reducing mortality due to diarrhea has been documented. Reports indicate that the total annual number of deaths caused by diarrhea among children under age 5 reduced by 61% from 2000 to 2019 [3]. This reduction has been attributed to the expanded use of ORS for diarrhea treatment [3]. In order to ensure availability and use of ORS at community and local levels, UNICEF and partners took the following actions: (i) Governments were encouraged to review and update their policy and regulation on ORS and zinc (ii) ORS and zinc were included in WHO’s priority medicines for mothers and children (iii) flavoured ORS for formulations were introduced into UNICEF’s supply catalogue (iv) UNICEF in conjunction with manufacturers introduced a 10.2g/0.5 litre sachet package for ORS treatment (v) UNICEF established long-term arrangements with different ORS and zinc certified manufacturers in Africa, Asia and Europe and (vi) wholesalers and distributors encouraged to include ORS and zinc in their supply chains [4].

The proportions of under-five children with diarrhea who received ORS in sub-Saharan Africa as at 2021 was 36% [2]. This rate is far below what was recorded for developing countries [4]. It is also indicated that there are gaps in access to ORS between the richest and poorest households in most regions, including sub-Saharan Africa [5]. Factors that influence use of ORS for children with diarrhea include household wealth, exposure to media, health-seeking behaviour, recognition of severity of signs of dehydration, availability of ORS, awareness of ORS, residence, mother’s age, age, health insurance, among others [6–13].

As a result of the high rate of under-five deaths linked to diarrhea in sub-Saharan Africa and the low rate of ORS use, it has become necessary to investigate more on the factors that are contributing to this poor use of ORS in the region. However, most of the studies on ORS use were based on a specific country or locality at a specific time (with only a few considering more than one country) which does not allow for a broader perspective of the phenomenon. In such case, there is a need for a multi-country approach. This study aimed at filling this gap by assessing prevalence of ORS use and examining the factors that are associated with ORS use for children with diarrhea using data sets from 31 countries in sub-Saharan Africa.

## Methods

### Data

This study used data from Demographic and Health Surveys (DHS) involving 31 countries in sub-Saharan Africa. The surveys are cross-sectional and comprise information relating to population and health characteristics of women and children in the selected countries. These surveys were conducted between 2010 and 2018 and only the most recent surveys were considered for the countries. Data from all the countries were merged in order to have a single data set for the sub-region. Sampling procedure in the DHS involved two-stage cluster sampling method. In the first stage, enumeration areas (EAs) were selected from rural and urban areas using probability proportional to size. In the second stage, households were selected from each enumeration area. In the selected households, women of reproductive age (15–49 years) were interviewed. Standardized questionnaires were used to obtain information from these women. Some of the variables covered in the questionnaires include reproductive history, background characteristics, antenatal care, delivery care, immunization, childhood diseases, nutrition, health-seeking practice, HIV, domestic violence, fertility, and mortality.

### Outcome variable

The outcome variable in this study is the use of ORS **for** the treatment of diarrhea among under-five children. Mothers were first asked if their children had diarrhea at any time during the two-week period preceding the survey. If the mother’s response indicated that the child had diarrhea, she was then asked the action she took to treat the child. Specifically, such women were asked if they used **ORS for the treatment of their children.**

### Independent variables

The following independent variables were used in the study: mother’s age, education, household wealth, residence, media exposure, antenatal care attendance, sex of child, child’s age, birth order, child’s size at birth, number of under-five children living in household and source of water. Mother’s age was expressed as 15–24 years, 25–34 years and 35 years and above. Education was divided into none (for those with no education), primary and secondary/higher. Household wealth has five categories namely poorest, poorer, middle, richer and richest. This was arrived at by asking households the assets they possessed. Such assets include radio, television, car, bicycle, animals, farmland, housing features like roofing/flooring materials, toilet, and water source. These items were assigned using component analysis. The scores were thereafter aggregated resulting in an overall index. For easy classification, the index was expressed in five quintiles as poorest, poorer, middle, richer and richest. Residence was grouped as urban and rural. Media exposure was arrived at by defining women who were exposed to newspaper, radio or television as being exposed while those who were not exposed to any of the media outlets were defined as not exposed. Women who attended antenatal clinic when they were pregnant were defined as attended and those who did not attend were defined as never attended. Sex of child was described as male and female while child’s age was grouped into three as 0–11 months, 12–23 months and 24–59 months. Birth order was categorized into first order birth, second order birth and third or higher order birth. Child’s size at birth was expressed as large, average and small. The following categories were assigned to number of under-five children in household; at most 2 children, 3–4 children and 5 or more children. Source of water was grouped as improved and non-improved.

### Statistical analysis

The first stage of analysis involved pooling the data sets of the 31 countries together in order to have a single data set for sub-Saharan Africa. After this, weighting factor was used to weight the data set with the aim of removing the effects of over-reporting and under-reporting in the surveys. This was carried out by calculating the weighting factor ($\frac{v005}{1000000}$) and applying same to the data set through *svyset* command. Chi-Square test was then used to examine the association between ORS use among children and all the independent variables. Variables that were found to be significant at this level (denoted by p-values) were moved into the multivariable analysis. In the multivariate analysis, binary logistic regression was applied and odds ratios and confidence intervals were thereafter obtained for easy interpretations. Level of significance (*α*) was expressed at 0.05 and 0.001. All the analyses were carried out using Stata 14 statistical package.

## Results

### Sample characteristics

The study involved 30,102 under-five children who experienced diarrhea in two weeks preceding the surveys in sub-Saharan Africa. While 38% of the children used oral rehydration solution, 62% did not use it. Table 1 shows that countries with the highest percentage of children who used ORS include Namibia (71.8%), Zambia (66.4%) and Malawi (63.8%). Results in Table 2 show that use of ORS was most common among children whose mothers were 15–24 years of age (39%), had secondary or higher education (45%), exposed to media (40.8%) and attended antenatal care (41.1%). Children from richest households (43%), children age 12–23 months (42%), children of first order birth (40%) and those who were of average size at birth (39%) had the highest prevalence of oral rehydration solution use. Significant proportions of children who received ORS during their diarrhea episode were found among households with 2 or less under-five children (39%) and households with improved water source (40%).

**Table 1:** Year of survey, number of children and use of oral rehydration solution in Sub-Saharan Africa.

Country	Year of survey	Number of children	Children with diarrhea who used oral rehydration solution
			(%)
Angola	2015–2016	972	41.5
Benin	2017–2018	1,327	21.6
Burkina Faso	2010	1,065	21.0
Burundi	2016–2017	1,336	34.8
Cameroon	2011	1,133	19.2
Chad	2014–2015	2,151	19.7
Comoros	2012	471	38.4
Congo	2011–2012	793	22.7
Cote d’Ivoire	2011–2012	648	16.3
Democratic Republic of Congo	2013–2014	1,468	35.5
Ethiopia	2016	1,076	36.1
Gabon	2012	638	23.7
Gambia	2013	666	61.1
Ghana	2014	336	48.8
Guinea	2018	419	46.7
Kenya	2014	2,906	51.9
Lesotho	2014	168	47.6
Liberia	2013	827	60.2
Malawi	2015–2016	1,109	63.8
Mali	2018	1,565	22.0
Namibia	2013	395	71.8
Niger	2012	790	43.7
Nigeria	2018	1,485	38.8
Rwanda	2014–2015	459	28.3
Senegal	2010–2011	878	21.8
South Africa	2016	158	44.3
Tanzania	2015–2016	1,111	44.1
Togo	2013–2014	537	21.5
Uganda	2016	1,016	45.2
Zambia	2018	1,415	66.4
Zimbabwe	2015	887	41.7

**Table 2:** Relationship between use of oral rehydration solution for children with diarrhea and independent variables in Sub-Saharan Africa.

Variables	Children with diarrhea treated with oral rehydration solution			p-value
Mother’s age	No N (%)	Yes N (%)	Total N (%)	
	18,711 (62.2)	11,391 (37.8)	30,102 (100.0)	
15–24 years	6,290 (61.3)	3,964 (38.7)	10,254 (100.0)	
25–34 years	8,642 (62.0)	5,289 (38.0)	13,931 (100.0)	
35+ years	3,779 (63.9)	2,138 (36.1)	5,917 (100.0)	0.006
Education				
None	8,342 (69.6)	3,649 (30.4)	11,991 (100.0)	
Primary	6,444 (58.5)	4,566 (41.5)	11,010 (100.0)	
Sec/higher	3,925 (55.3)	3,176 (44.7)	7,101 (100.0)	<0.001
Household wealth				
Poorest	5,558 (66.0)	2,861 (34.0)	8,419 (100.0)	
Poorer	4,367 (63.2)	2,542 (36.8)	6,909 (100.0)	
Middle	3,487 (60.5)	2,277 (39.5)	5,764 (100.0)	
Richer	3,059 (60.2)	2,020 (39.8)	5,079 (100.0)	
Richest	2,240 (57.0)	1,691 (43.0)	3,931 (100.0)	<0.001
Residence				
Urban	5,063 (58.0)	3,660 (42.0)	8,723 (100.0)	
Rural	13,648 (63.8)	7,731 (36.2)	21,379 (100.0)	<0.001
Media exposure				
Not exposed	7,658 (67.0)	3,777 (33.0)	11,435 (100.0)	
Exposed	11,030 (59.2)	7,595 (40.8)	18,625 (100.0)	<0.001
ANC attendance				
Never attended	2,314 (79.5)	598 (20.5)	2,912 (100.0)	
Attended	12,902 (58.9)	9,004 (41.1)	21,906 (100.0)	<0.001
Sex of child				
Male	9,734 (62.0)	5,972 (38.0)	15,706 (100.0)	
Female	8,977 (62.4)	5,419 (37.6)	14,396 (100.0)	0.496
Child’s age				
0–11 months	5,131 (65.9)	2,661 (34.2)	7,792 (100.0)	
12–23 months	5,797 (58.3)	4,152 (41.7)	9,949 (100.0)	
24–59 months	7,783 (63.0)	4,578 (37.0)	12,361 (100.0)	<0.001
Birth order				
First order birth	10,202 (60.5)	6,661 (39.5)	16,863 (100.0)	
Second order birth	5,786 (63.2)	3,377 (36.8)	9,163 (100.0)	
Third or higher order birth	2,723 (66.8)	1,353 (33.2)	4,076 (100.0)	<0.001
Child’s size at birth				
Large	6,648 (63.4)	3,845 (36.6)	10,493 (100.0)	
Average	7,558 (61.2)	4,799 (38.8)	12,357 (100.0)	
Small	3,505 (65.0)	1,888 (35.0)	5,393 (100.0)	<0.001
Number of under-five children in household				
≤2 children	13,029 (60.6)	8,486 (39.4)	21,515 (100.0)	
3–4 children	4,672 (65.5)	2,463 (34.5)	7,135 (100.0)	
≥5 children	1,010 (69.6)	442 (30.4)	1,452 (100.0)	<0.001
Source of water				
Improved	11,469 (59.9)	7,683 (40.1)	19,152 (100.0)	
Non-improved	6,851 (66.3)	3,482 (33.7)	10,333 (100.0)	<0.001

p-value = level of significance.

### Factors associated with oral rehydration solution use

Table 3 shows that women who had primary and secondary or higher education are 1.43 times and 1.63 times respectively more likely to use ORS for their children than their counterparts who have no education. Women who are exposed to media are 1.17 times more likely to use ORS for their children compared to women who are not exposed to media. The odds of using ORS for children with diarrhea increased by a factor of 2.33 for women who attended antenatal care during their pregnancy period. Children who are 12–23 months old and those who are 24–59 months old are 1.46 times and 1.29 times respectively more likely to receive ORS than children who are 0–11 months old. Children who were of average size at birth are 1.08 times more likely to receive ORS compared to their counterparts who were large in size at birth. The odds of receiving ORS during diarrhea episode increased by factors of 1.28 and 1.18 respectively for children who are from households with 2 or less under-five children and improved water source.

**Table 3:** Logistic regression of factors associated with oral rehydration solution use in the treatment of diarrhea among children in Sub-Saharan Africa.

Variable	Received oral rehydration solution	
	Odds ratio	95% Confidence interval
Mother’s age		
15–24 years	1	
25–34 years	1.04	0.96–1.14
35+ years	1.06	0.93–1.20
Education		
None	1	
Primary	1.43**	1.32–1.57
Sec/higher	1.63**	1.47–1.81
Household wealth		
Poorest	1	
Poorer	1.00	0.91–1.11
Middle	1.02	0.92–1.14
Richer	1.01	0.89–1.14
Richest	1.03	0.89–1.19
Residence		
Urban	0.95	0.85–1.06
Rural	1	
Media exposure		
Not exposed	1	
Exposed	1.17**	1.07–1.27
ANC attendance		
Never attended	1	
Attended	2.33**	1.08–1.27
Child’s age		
0–11 months	1	
12–23 months	1.46**	1.35–1.59
24–59 months	1.29**	1.19–1.42
Birth order		
First order birth	1.08	0.94–1.24
Second order birth	1.11	0.98–1.25
Third or higher order birth	1	
Child’s size at birth		
Large	1	
Average	1.08*	1.00–1.17
Small	0.97	0.88–1.08
Number of under-five children in household		
≤2 children	1.28*	1.06–1.54
3–4 children	1.21	0.99–1.48
≥5 children	1	
Source of water		
Improved	1.18**	1.09–1.29
Non-improved	1	
**Goodness of fit**		
F-adjusted test statistic	1.529	
Prob > F	0.132	

Level of significance at

*p<0.05

**p<0.001.

## Discussion

This study shows that prevalence of oral rehydration solution use in the 31 countries considered is 38% with countries such as Namibia, Zambia and Malawi having the highest rates. Education plays an important role in ORS use as women with primary and secondary or higher education are more likely to use ORS for their children compared to uneducated women. This is in line with other studies that have underscored the role of maternal education in ORS use. This may be attributed to the assumption that educated women are more equipped with information about childcare and child welfare than uneducated women. For instance, it was emphasized in previous studies that educated mothers understand better the complications of childhood diarrhea and as a result would seek early and appropriate treatment for their children [14–18].

Results also show that women who are exposed to media are more likely to use ORS for their children than their counterparts who are not exposed to media. Media exposure provides an opportunity for women to have access to information on various issues including child health [12,19,20]. Child health programmes on radio and television contribute immensely to improved awareness of mothers on childcare including therapies that could be administered at home. Antenatal care attendance also shows a significant association with ORS use. Women who attended antenatal care are more likely to use ORS for their children than women who did not attend antenatal care [21]. Apart from adequate care which women attending antenatal clinic receive, they are also exposed to health talks which prepare them for proper child care. Such opportunities, more often than not, elude women who do not attend antenatal care. Results further show that children age 12–23 months and 24–59 months are more likely to receive ORS during diarrhea episode than children who are 0–11 months old. This may be due to the fact that diarrhea is common among older children compared to infants [22]. In addition, breastfeeding, which has an important role in prevention of diarrhea, is more common among children 0–11 months. With respect to size of child at birth, children who were of average size at birth are more likely to use ORS than children who were large in size at birth. This indicates that size of child at birth is a factor influencing use of ORS. The preference of average-size children over large-size children in the use of ORS may be linked to the notion among women that large-size children are stronger than other categories of children. Results by number of under-five children in households show that children from households with 2 or less children are more likely to receive ORS than children from households with 2 or more children. This may be attributed to the fact that women in households with 2 or more children share their time with more children which may lead them into paying little attention to the plight of such children [23]. Relationship between water source and ORS use shows that children from households with improved water source are more likely to receive ORS compared to children from households with non-improved water source [14]. This may be explained by the assumption that that since water is involved in the preparation of ORS, women from households with improved water source are more inclined to prepare the solution.

It is likely that some of the factors above could be more important than others in influencing ORS use and some could relate to one another. Household with improved water source is likely to have higher income and at the same time having higher education. Having higher education could also enhance information-seeking behaviour leading to educated women being more exposed to media than uneducated women. In the same vein, antenatal care attendance may be influenced by women’s level of education and exposure to information.

### Strengths and weaknesses

Some of the shortcomings in this study were: cause and effect could not be established as a result of the cross-sectional nature of the data; severity of diarrhea as a determinant of ORS use is another variable that this study did not include and the study could not also determine reasons why there were differences in ORS use among the countries considered. Despite these shortcomings, our study has provided a robust information on the use of ORS for diarrhea treatment among children in sub-Saharan Africa. The use of multi-country approach afforded the study the advantage of having a large sample size which eventually contributed to reliability and generalizability of its findings.

## Conclusion

This study has revealed that the prevalence of ORS use for diarrhea treatment among under-five children in sub-Saharan Africa is 38% which is even lower than the rate recorded in developing countries as a whole (44%). It has also revealed the factors associated with this low ORS use. Such factors include mother’s education, media exposure, antenatal care attendance, child’s age, child’s size at birth, number of under-five children living in household and water source. Appropriate measures need to be put in place to increase rate of ORS use for diarrhea treatment among children in the sub-region. Such measures should involve intensifying efforts on girl-child education to increase literacy level among women. This requires government involvement in the provision of educational facilities not only at the national level but also at local level to reach people at the grass root. There is also the need to increase women’s media exposure so that they can benefit from enlightenment programmes relating to maternal and child health. Ensuring that there is an increase in the number of women that attend antenatal care during pregnancy is another area that should be considered. This should involve adequate participation of health workers through the Ministry of Health. Awareness programmes on importance of early and adequate attendance of antenatal care should be regularly organized for women at community level. Also, government at local and national levels should ensure that more allocation is allotted to provision of amenities such as water as a way of assisting people to obtain water from good source. Governments at national level should ensure that a tracking system is put in place to monitor the progress in the use of ORS across the length and breadth of each country. Above all, further studies should focus on examining the effects of severity of diarrhea on ORS use and the factors that determine differences in ORS use among countries in sub-Saharan Africa.

## Supporting information

S1 File (DOCX)

## Abbreviations

aOR: Adjusted Odds Ratio
CI: Confidence Interval
DHS: Demographic and Health Survey
ORS: Oral Rehydration Solution
UNICEF: United Nations International Children’s Emergency Fund
WHO: World Health Organization
